# HuR Affects Proliferation and Apoptosis of Chronic Lymphocytic Leukemia Cells via NF-*κ*B Pathway

**DOI:** 10.1155/2020/1481572

**Published:** 2020-08-27

**Authors:** Kai Xiao, Lin Yang, Xinfeng Gao, Ying An, Wei Xie, Guo Jingquan

**Affiliations:** ^1^Department of Foot & Ankle Surgery, Wuhan Fourth Hospital, Puai Hospital, Tongji Medical College, Huazhong University of Science and Technology, Wuhan, China; ^2^Department of Allergy, Tongji Hospital of Tongji Medical College of HUST, Wuhan, China; ^3^Department of Orthopedic, Wuhan Fourth Hospital, Puai Hospital, Tongji Medical College, Huazhong University of Science and Technology, Wuhan, China

## Abstract

**Objective:**

To investigate the effects of HuR protein on the treatment of chronic lymphocytic leukemia (CLL).

**Methods:**

LCL lymphoblast cells and B lymphocytes were subjected to HuR overexpression (OV) or interference (IV). Western blot was used to observe the protein expression of human tumor necrosis factor-associated factor 1 (TRAF1), human inhibitor of nuclear factor kappa-B kinase *α* (IKK-*α*), NF-*κ*B-inducing kinase (NIK), and p52. Flow cytometry was performed to evaluate apoptosis, and the mRNA expression of TRAF1 was examined by quantitative reverse transcription polymerase chain reaction. Immunofluorescence was carried out to visualize the expression of HuR, and the relationship between HuR and TRAF1 was observed by pull-down test. Cell sensitivity to chlorambucil (CLB) and fludarabine (Flu) was assessed by Cell Counting Kit-8.

**Results:**

The expression of HuR and TRAF1 in LCLs was significantly increased compared to that in B lymphocytes. Compared with the control, HuR OV significantly increased the expression of TRAF1 (*P* < 0.05), whereas it was significantly decreased in the IV group (*P* < 0.05). HuR can bind to TRAF1 directly, and the binding rate is positively correlated with HuR expression. After inhibiting HuR, the expression of TRAF1, IKK-*α*, NIK, p52, pro-Caspase 3, and PARP was significantly upregulated in LCLs and B lymphocytes (*P* < 0.05), while Caspase 3 was downregulated (*P* < 0.05). Compared with the control, the proliferation of LCLs and B lymphocytes treated by CLB and Flu decreased significantly after HuR blockade (*P* < 0.05).

**Conclusion:**

HuR may be a key protein regulating CLL resistance. After inhibiting HuR, inflammatory response and apoptosis were significantly increased, and the cell sensitivity to CLB and Flu increased, suggesting that inhibiting HuR activity may be a potential strategy to solve the problem of drug resistance in CLL cells.

## 1. Introduction

Leukemia is a malignant clonal disease of hematopoietic stem cells, with fever, anemia, and lymphadenopathy as its main clinical symptoms. Chronic lymphocytic leukemia (CLL) is a subtype of leukemia that originates from hematopoietic tissue. Tumor cells are monoclonal B lymphocytes with similar morphology to that of normal mature small lymphocytes and mainly exist in blood, bone marrow, and lymphoid tissue, with poor prognosis. Nowadays, the main treatment methods of CLL was chemotherapy. Commonly used drugs include chlorambucil (CLB), fludarabine (Flu), and glucocorticoids. However, the long-term use of chemotherapeutic drugs reduces the sensitivity of tumor cells to the drugs, resulting in a decline in drug efficacy. There is presently no ideal method of CLL treatment. The RNA-binding protein HuR is a member of the embryonic lethal abnormal vision gene family that can affect the expression of target genes by regulating the stability and translation efficiency of target mRNA. HuR participates in the regulation of biological activities and is an important mediator of cell division, cell senescence, immune cell activation, and other vital activities closely related to inflammation and tumorigenesis [1, 2]. Danilin et al. showed that HuR inhibited tumor cell apoptosis and promoted tumor cell proliferation and migration, and that inhibition of HuR activity resulted in obvious antitumor properties [3, 4]. Tumor-necrosis factor (TNF)-*α* is a potent antitumor, proapoptotic, and proinflammatory cytokine in vivo. HuR protein specifically binds to the 3′-untranslated region of TNF-*α* and is closely related to various diseases [5–7]. In this study, we investigated the interaction between TNF-*α* and HuR by exploring the effect of TNF-*α* on the apoptosis and inflammatory response of lymphoblast cells and B lymphocytes after inhibiting the expression of HuR. We aim to provide a possible solution to CLB and Flu resistance in the clinical treatment of CLL.

## 2. Materials and Methods

### 2.1. Lymphoblast Cells and B Lymphocytes

The lymphoblast cell line LCLs and B lymphocytes were obtained from the American Type Culture Collection (Virginia, USA) and cultured in RPMI 1640 medium (SH30809.01B, Hyclone, Utah, USA) supplemented with 10% fetal bovine serum (10270-106, Gibco, CA, USA) in an atmosphere containing 5% CO_2_ and 95% air at 37°C for 24 hours. The medium was replaced every 24 hours, and the cells were subcultured or cryopreserved when they reached 70–80% confluence.

### 2.2. Vector Construction and Transfection

The HuR sequences were forward, 5′-GCTCTAGAATGTCTAATGGTTATGAAGAC-3′ and reverse, 5′-CGGGATCCTTATTTGTGGGACTTGTTGGT-3′. The cDNA of the human HuR gene was inserted into the pcDNA3.1-HuR vector (addgene, NC, USA). The restriction sites were Xho I and BamH I. After the digested PCR gene fragments and linearized vector were connected at 16°C overnight, the acquired HuR overexpression vectors were transformed into competent DH5*α* cells. The target plasmids were extracted from the bacterial liquid according to the instructions provided.

HuR interference (5′-GAGTGAAGGAGTTGAAACT-3′) vectors were constructed using pSuper-neo-HuRsiRNA via a standard procedure including target gene identification, design, preparation, and transfection of pcDNA3.1. The vectors were then transfected according to the instructions of Lipofectamine® RNAiMAX (13778030, Invitrogen, CA, USA).

The LCLs and B lymphocytes were each divided into four groups: control (not subjected to transfection), EV (empty pcDNA3.1-HuR vector), OV (HuR overexpression vector), and IV (HuR interference vector).

### 2.3. Immunofluorescence

Cells were fixed with 4% paraformaldehyde for 10 min at room temperature, permeabilized with 0.3% Triton X-100, and blocked with 5% bovine serum albumin. The cells were then incubated with anti-HuR (1 : 150, ab200342, abcam, UK) overnight at 4°C, followed by incubation with Alexa Fluor 488 secondary antibody (1 : 200, PAB160027, Bioswamp, Wuhan, China) for 30 min at room temperature and counterstaining with DAPI to identify the nuclei. Images were captured with a fluorescence microscope (Nikon, Tokyo, Japan).

### 2.4. Western Blot

Protein extracts (10 *μ*g) prepared from LCLs or B lymphocytes were separated by 12% sodium dodecyl sulfate-polyacrylamide gel electrophoresis and transferred to polyvinylidene fluoride membranes (Millipore, Wisconsin, USA). The membranes were blocked with 5% milk in Tris-buffered saline (pH 7.6) containing 0.1% Tween 20 and incubated overnight at 4°C with specific primary antibodies against the following proteins: TNF receptor-associated factor 1 (TRAF1, 1 : 1000, ab203316, abcam, Cambridge, UK), I*κ*B kinase (IKK)-*α* (1 : 10000, ab109749, abcam), NF-*κβ*-inducing kinase (NIK, 1 : 500, ab203568, abcam), P52 (1 : 2000, ab129097, abcam), pro-caspase 3 (1 : 1000, ab32150, abcam), caspase 3 (1 : 500, ab49822, abcam), poly (ADP-ribose) polymerase (PARP, 1 : 2000, ab74290, abcam), and *β*-actin (1 : 5000, 66009-1-Ig, Proteintech, USA). After three washes with PBS/Tween 20, the membranes were incubated with horseradish peroxidase-conjugated secondary goat antirabbit IgG (1 : 20000, PAB160011, Bioswamp) for 2 h at room temperature. Protein bands were visualized by enhanced chemiluminescence color detection (Tanon-5200, TANON, Shanghai, China) and analyzed using AlphaEase FC gel image analysis software.

### 2.5. Quantitative Reverse Transcription Polymerase Chain Reaction (qRT-PCR)

Cells were extracted using Trizol reagent according to the manufacturer's procedures; cDNA was synthesized using a reverse transcriptase kit (TAKARA, Osaka, Japan). qPCR was performed with a real-time system (BIO-RAD, CA, USA) using the SYBR Green PCR Kit (KM4101, KAPA Biosystems, Massachusetts, USA). Each qPCR reaction (95°C, 3 min for denaturation; 95°C, 5 s and 56°C, 10 s and 72°C, 25 s for 39 cycles; 65°C, 5 s and 95°C, 50 s) was performed in duplicate. The results were analyzed by the 2^-*△△*Ct^ method. The primers were designed and configured by Nanjing Kingsy Biotechnology Co., Ltd. and were listed in Table 1.

### 2.6. Pull-down Test

10^5^ cells were lysed by 2 mL of cell lysis buffer, centrifuged at 2500 rpm for 5 minutes, and incubated with 2 *μ*g of anti-HuR (1 : 150, ab200342, abcam) overnight at 4°C. Then 20 *μ*l of Protein A+G Sepharose (P001-2, QiHai, China) was added and centrifuged at 2500 rpm, and the cells were resuspended with DEPC water (PAB180005, Bioswamp, China). cDNA was synthesized using a reverse transcriptase kit (TAKARA) and amplified using a PCR amplifier (T100-Thermal Cycler, Bio-RAD). The data were analyzed by qbase plus. The primers were designed and configured by Nanjing Kingsy Biotechnology Co., Ltd. (Table 1).

### 2.7. Flow Cytometry

10^5^ cells were cultured for 24 h and harvested. Then, 1 ml of precooled PBS was added to the cells and centrifuged at 1000 × g. After the addition of 10 *μ*l of Annexin V-fluorescein isothiocyanate (FITC) and 10 *μ*L of propidium iodide, the cell samples were analyzed by flow cytometry (Beckman Coulter, USA).

### 2.8. Cell Counting Kit-8 (CCK8)

Cells were seeded in a 96-well plate at 3 × 10^3^ cells/ml using 1640 medium containing 10% fetal bovine serum. Cells were treated with CLB (20 *μ*mol/l) and Flu (10 *μ*mol/l) either in the presence or absence of 1 mM AICAR, an inhibitor of HuR, for 24 h. To evaluate cell proliferation, 10 *μ*l of CCK8 solution was added to each well, and the cells were cultured at 37°C for 4 h. The optical density was measured using a plate reader (Multiskan FC, Thermo, USA) at 450 nm.

### 2.9. Statistical Analysis

The data are expressed as the mean ± standard deviation (SD). Data comparison was performed by *t*-tests and one-way analysis of variance using SPSS 22 statistical software. *P* < 0.05 was considered statistically significant.

## 3. Results

### 3.1. Expression of HuR and TRAF1 in LCLs and B Lymphocytes

**Figure 1:**
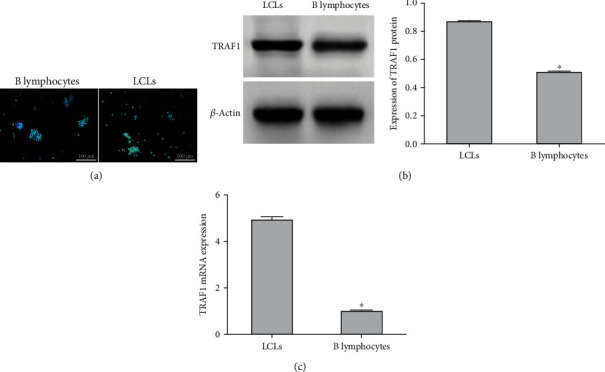
Expression of HuR and TRAF1 in LCLs and B lymphocytes. (a) HuR protein expression was measured by immunofluorescence (scale bar = 100 *μ*m); green and blue represent HuR and nucleus, respectively. (b) Western blot of the protein expression of TRAF1 in LCLs and B lymphocytes. (c) qRT-PCR of the mRNA expression of TRAF1 in LCLs and B lymphocytes. The results are presented as the mean ± SD, *n* = 3. ^∗^*P* < 0.05 vs. LCLs.

Immunofluorescence was performed to detect the protein expression of HuR in LCLs and B lymphocytes. The protein expression of HuR in LCLs was increased compared to that in B lymphocytes (Figure 1(a)). Western blot and qRT-PCR were carried out to examine the protein and mRNA expression of TRAF1, respectively. As shown in Figures 1(b) and 1(c), the protein and mRNA expression of TRAF1 in LCLs were significantly higher than those in B lymphocytes (*P* < 0.05).

### 3.2. HuR Binds Directly to TRAF1 and Promotes TRAF1 Protein Expression

**Figure 2:**
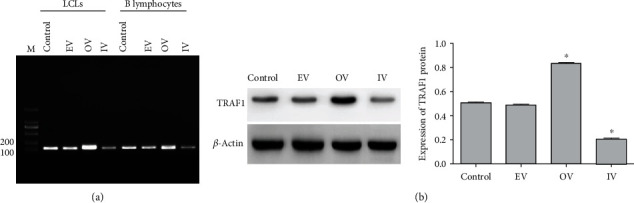
Relationship between HuR and TRAF1 was observed by pull-down test and western blot. (a) Relationship between HuR and TRAF1 was observed by pull-down test. (b) Expression of TRAF1 protein was measured by western blot. Control, no transfection; EV, empty pcDNA3.1-HuR vector; OV, HuR overexpression vector; IV, HuR interference vector. The results are presented as the mean ± SD, *n* = 3. ^∗^*P* < 0.05 vs. control.

The relationship between HuR and TRAF1 was investigated using a pull-down assay. We showed that HuR bound directly to TRAF1, and the binding rate of HuR-OV LCLs appeared to be higher than that of HuR-OV B lymphocyte (Figure 2(a)). Subsequently, the protein expression of TRAF1 was detected by western blot, as shown in Figure 2(b). Compared with the control group, TRAF1 expression was significantly increased in the OV group (*P* < 0.05), while that in IV the group decreased significantly (*P* < 0.05).

### 3.3. TNF-*α* Promotes Inflammatory Response and Apoptosis in LCLs and B Lymphocytes after Inhibiting HuR Expression

**Figure 3:**
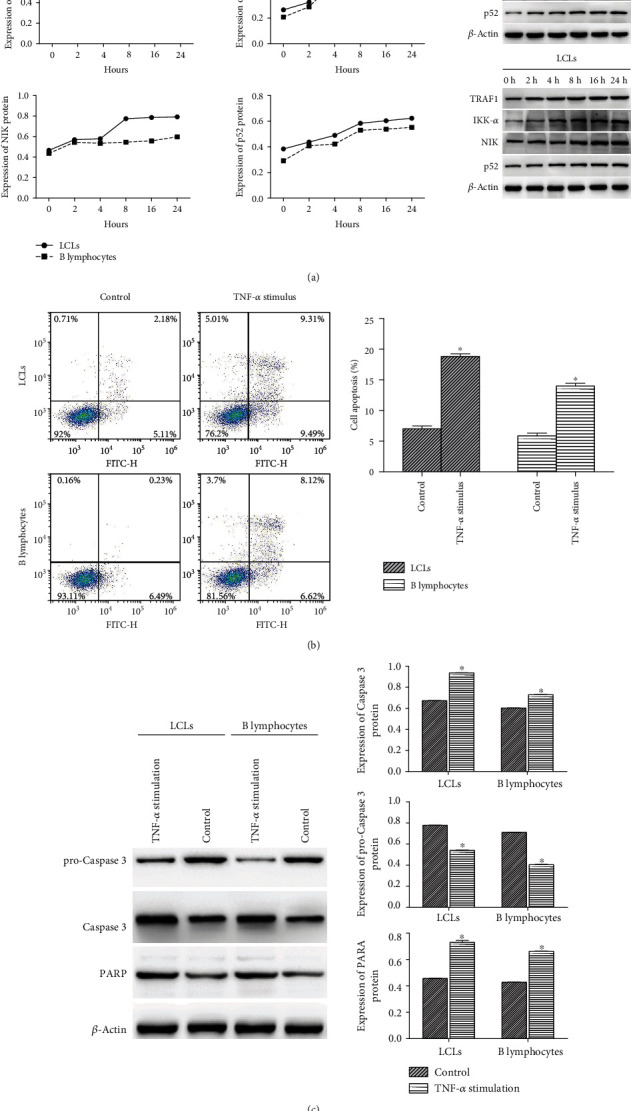
Effect of TNF-*α* on inflammatory response and apoptosis of LCLs and B lymphocytes. (a) Protein expression of TRAF1, IKK-*α*, NIK, and p52 was measured by western blot. (b) Flow cytometry analysis of apoptosis. (c) Protein expression of pro-Caspase 3, caspase 3, and PARP was measured by western blot. The results are presented as the mean ± SD, *n* = 3. ^∗^*P* < 0.05 vs. control.

LCLs and B lymphocytes were incubated with 1 mM AICAR (HuR inhibitor) for 4 h to block HuR expression. After stimulation with 50 ng/ml TNF-*α*, the effect of TNF-*α* activation on inflammatory response and apoptosis was observed in LCLs and B lymphocytes. As shown in Figure 3(a), the expression of TRAF1, IKK-*α*, NIK, and p52 increased over time and reached stable expression levels after 8 h of TNF-*α* treatment. Flow cytometry revealed that the percentage of apoptosis was significantly increased by TNF-*α* stimulation (*P* < 0.05) in both LCLs and B lymphocytes (Figure 3(b)). We further examined the activity of apoptosis-related proteins. Compared with control cells, those stimulated with TNF-*α* showed increased protein expression of Caspase 3 and PARP (*P* < 0.05) and decreased protein expression of pro-caspase 3 (*P* < 0.05) (Figure 3(c)).

### 3.4. HuR Inhibition Increased Sensitivity of LCLs and B Lymphocytes to CLB and Flu

**Figure 4:**
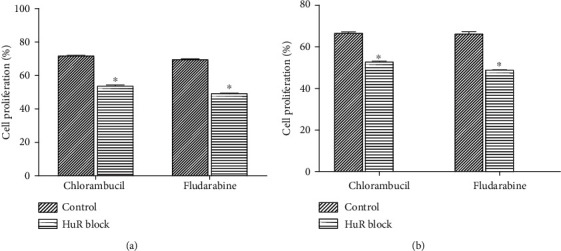
Proliferation rate of LCLs and B lymphocytes after chlorambucil or fludarabine intervention was detected by CCK8 assay. (a) Proliferation rate of LCLs. (b) Proliferation rate of B lymphocytes. The results are presented as the mean ± SD, *n* = 3. ^∗^*P* < 0.05 vs. control.

We next evaluated whether inhibition of HuR had an effect on the sensitivity of LCLs and B lymphocytes to CLB and Flu. We demonstrated that inhibition of HuR in both LCLs (Figure 4(a)) and B lymphocytes (Figure 4(b)) resulted in a decrease in survival when the cells were treated with CLB or Flu (*P* < 0.05). The observations suggested that the blockade of HuR increased the sensitivity of LCLs and B lymphocytes to CLB and Flu.

## 4. Discussion

Drug resistance is a common problem in the treatment of CLL and is mainly manifested by a decrease in the sensitivity of cancer cells to chemotherapeutic drugs after several courses of treatment. As a result, the efficacy of chemotherapeutic drugs decreases gradually, which is an important reason for the failure of chemotherapy. HuR is widely expressed in various tissues of the body and has three RNA recognition motif (RRM) domains. RRM1 and RRM2 at the N terminal are involved in binding to target mRNA molecules. HuR specifically binds to the AU-rich element in the 3′-UTR of its target gene, which can upregulate the expression of the target gene by improving its stability and/or translation efficiency [8]. Kullmann et al. found that HuR can also negatively regulate target genes through specific mechanisms by inhibiting the translation of related mRNA [9]. Up to now, HuR has been confirmed to regulate the expression of cyclin A, c-fos, vascular endothelial growth factor, TNF-*α*, *β*-catenin, c-Myc, cyclooxygenase-2, myogenin, MyoD, granulocyte-macrophage colony-stimulating factor, interleukins (ILs), p21, p27, p53, and heat shock protein 70. This occurs through posttranscriptional regulation, which is an important mediator of cell response to stress stimuli, cell division, activation of immune cells, and muscle differentiation [10–13]. Under inflammatory conditions, HuR can bind to the mRNA of proinflammatory factors such as TNF-*α* and IL-4 to upregulate their protein expression [14]. However, Katsanou et al. found that overexpression of the HuR gene inhibited inflammation. Although the binding of HuR protein to TNF-*α* mRNA can improve the stability of HuR, the translation of TNF-*α* is inhibited [15]. Yiakouvaki et al. confirmed that the concentration of TNF-*α*, IL-6, IL-12, and other proinflammatory cytokines in the serum of HuR-deficient transgenic mice increased significantly after external stimulation [1], prompting that HuR has both proinflammatory and anti-inflammatory effects.

In this study, the protein expression of HuR and TRAF1 in LCLs was higher than that in normal human B lymphocytes. It is suggested that the expression of HuR and TRAF1 is increased significantly in the pathological state. We further studied the interaction between HuR and TRAF1 and found that HuR binds directly to TRAF1. The overexpression of HuR enhanced the protein expression of TRAF1, suggesting that the increased expression of TRAF1 in the pathological state might be caused by the upregulation of HuR. The members of the TRAF family are intracellular adaptor proteins that can directly or indirectly bind to a variety of TNFs, ILs, and toll-like receptors. They mediate signal transduction and play an important role in both the classical and nonclassical NF-*κ*B signaling pathways. TNF receptor 1 (TNFR1) and TNFR2 are both important receptors for TNF-*α*. TNFR1 has a “death domain” consisting of 80 conserved amino acids and can recruit TNFR1-associated death domain protein and bind directly to TNF-*α*. TNFR2 lacks this “death domain” and only recruits TRAF1 and TRAF2, binding to TNF-*α* through TNFR-related factors. Since TNFR2 lacks the death domain, the combination of TNF-*α* and TNFR2 does not promote apoptosis [16]. In this study, the increased protein expression of HuR enhanced the expression of TRAF1, and the increased expression of TRAF1 may promote the binding of TNF-*α* to TNFR2 while reducing the binding of TNF-*α* to TNFR1, thus reducing apoptosis. After inhibiting HuR, we observed that the expression of inflammation-related factors and apoptosis of LCLs and B lymphocytes increased significantly after TNF-*α* intervention, suggesting that inhibition of HuR accentuated the proinflammatory and proapoptotic effects of TNF-*α*. Previous studies have shown that both CLB and Flu promoted the expression of TNF-*α* [17, 18]. In this study, the sensitivity of LCLs and B lymphocytes to CLB and Flu was significantly increased when HuR was inhibited, suggesting that HuR may be a key protein in regulating cell resistance.

In conclusion, HuR may be the key protein regulating drug resistance in CLL cells. Inhibition of HuR can significantly improve the drug sensitivity of CLL cells to CLB and Flu and improve the clinical efficacy of these drugs.

## Figures and Tables

**Table 1:** Primer sequences.

Primer	Sequence (5′-3′)
TRAF1-F	ATCTGTCGCTCTTCATCG
TRAF1-R	CACGGTTGTTCTGGTCC

## Data Availability

The data used to support the findings of this study are available from the corresponding author upon request.
